# Application of Aquatic Insects (Ephemeroptera, Plecoptera And Trichoptera) In Water Quality Assessment of Malaysian Headwater

**DOI:** 10.21315/tlsr2017.28.2.11

**Published:** 2017-07-31

**Authors:** Suhaila Ab Hamid, Che Salmah Md Rawi

**Affiliations:** School of Biological Sciences, Universiti Sains Malaysia, 11800 USM Pulau Pinang. Malaysia

**Keywords:** Ephemeroptera, Plecoptera, Trichoptera, Water Quality Index, EPT Taxa Richness Index

## Abstract

The Ephemeroptera, Plecoptera and Trichoptera (EPT) community structure and the specific sensitivity of certain EPT genera were found to be influenced by water parameters in the rivers of Gunung Jerai Forest Reserve (GJFR) in the north of peninsular Malaysia. The scores of EPT taxa richness of >10 in all rivers indicated all rivers’ habitats were non-impacted, having good water quality coinciding with Class I and Class II of Malaysian water quality index (WQI) classification of potable water. The abundance of EPT was very high in Teroi River (9,661 individuals) but diversity was lower (22 genera) than Tupah River which was highly diverse (28 genera) but lower in abundance (4,263 individuals). The lowest abundance and moderate diversity was recorded from Batu Hampar River (25 genera). *Baetis* spp. and *Thalerosphyrus* spp., *Neoperla* spp. and *Cheumatopsyche* spp. were the most common genera found. Classification for all rivers using EPT taxa Richness Index and WQI gave different category of water quality, respectively. The WQI classified Tupah and Batu Hampar rivers into Class II and Teroi River (Class I) was two classes above the classification of the EPT taxa Richness Index.

## Abstract (ru)

Struktur komuniti Ephemeroptera, Plecoptera dan Trichoptera (EPT) dan sensitiviti khusus genera EPT tertentu didapati dipengaruhi oleh parameter air di dalam sungai Hutan Simpan Gunung Jerai (HSGJ) di utara Semenanjung Malaysia. Skala kekayaan taksonomi EPT > 10 di semua sungai menunjukkan bahawa semua habitat sungai tidak terjejas, yang mempunyai kualiti air yang baik bersamaan dengan klasifikasi Kelas I dan Kelas II indeks kualiti air Malaysia (WQI) bagi air minuman. Kelimpahan EPT adalah sangat tinggi di Sungai Teroi (9,661 individu) tetapi kepelbagaian lebih rendah (22 genera) daripada Sungai Tupah yang sangat pelbagai (28 genera) tetapi lebih rendah (4,263 individu). Keanekaragaman yang paling rendah dan sederhana telah dicatatkan dari Sungai Batu Hampar (25 genera). *Baetis* spp. Dan *Thalerosphyrus* spp., *Neoperla* spp. dan *Cheumatopsyche* spp. adalah genera yang paling biasa dijumpai. Klasifikasi untuk semua sungai yang menggunakan Indeks Kekayaan taxa EPT dan WQI memberikan kategori kualiti air yang berlainan. WQI mengklasifikasikan sungai Tupah dan Batu Hampar ke Kelas II dan Teroi River (Kelas I) adalah dua kelas di atas klasifikasi Indeks Kesejahteraan EPT.

## INTRODUCTION

In Asian inland aquatic ecosystem, various factors has threatened biodiversity which are related to human activities (Yule & Yong 2004; Gopal 2005; Dudgeon 2000; 2008). Being a nation with the highest consumption of water, freshwater resources in Malaysia especially streams and rivers became the ultimate importance to the country development by contributing up to 98% of the overall water consumption (DOE 2002). Unfortunately, few studies conducted in northern region of Peninsular Malaysia showed that many rivers were contaminated with pesticides, industrial wastes and heavy metals (DOE 2003; Al Shami *et al*. 2010; Salman *et al.* 2010). More research towards understanding the ecological and biological responses of rivers to pollution and development of assessment methods are urgently needed to protect these ecosystems (Yap 2005; Azrina *et al*. 2006; Che Salmah *et al.* 2007).

Most of the studies on benthic macroinvertebrates in relation to water quality evaluation have included analyses on community of Ephemeroptera, Plecoptera and Trichoptera (EPT) (Rosenberg & Resh 1993; Suhaila *et al.* 2011; Suhaila *et al*. 2014; Suhaila & Che Salmah 2014; Suhaila *et al*. 2016). EPT are very much intolerable to any presence of pollutants in the water bodies and thus EPT are crucial biological indicators in determining water quality of the river. In many instances, active anthropogenic activities nearby the river can have an effect on abundance and diversity of EPT (Wan Mohd Hafezul *et al*. 2016). Among the EPT, Plecoptera (stonefly) is the most sensitive to changes in water quality. Their low motility (Lazaridou-Dimitriadou 2002) and high heterogeneity suggests inevitable reactions towards changes in water quality from some members (Hellawell 1986). The presence of EPT species denotes that parameters in the habitat is within the tolerance limit of the species. Apart from Plecoptera, Trichoptera larvae and Ephemeroptera (mayflies) nymphs are also good bioindicators of the freshwater quality and ecological changes because most of the genera are only able to survive in rivers or streams with a good quality of water (Chapman 1996; Azrina *et al*. 2006; Suhaila *et al* 2014; Suhaila & Che Salmah 2014) and their distributions are strongly dictated by their tolerance to a set of environmental factors (Dudgeon 1984).

Communities of EPT are usually dominant in headwater rivers and the evaluation of water quality using these three insects orders are decent enough and satisfactorily accurate (Bonada *et al*. 2006; Suhaila *et al*. 2011, 2014). Therefore, this study was conducted to assess the quality of water through EPT assemblages in respective rivers and compared them with the conventional chemical classification of water quality exercised by the Malaysian Department of Environment.

## MATERIALS AND METHODS

### Study Area

**Figure 1:**
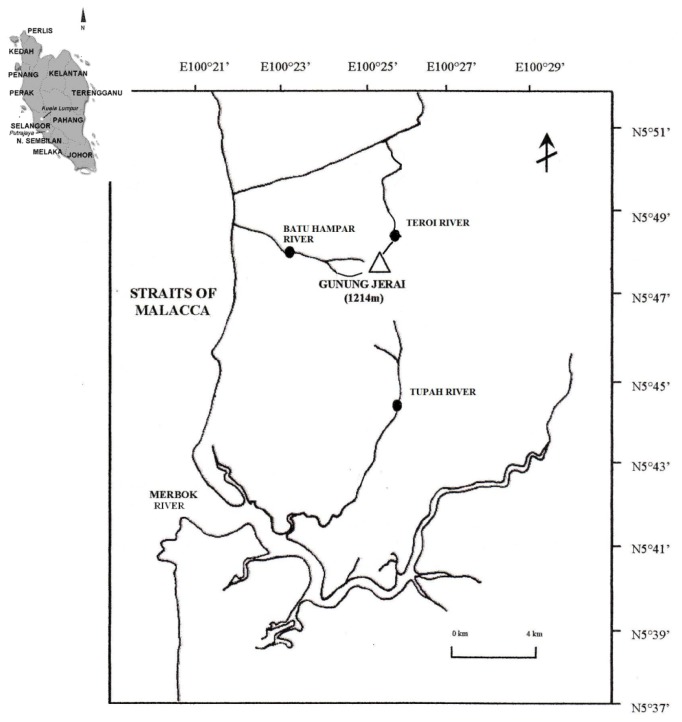
Location of sampling areas, Tupah, Batu Hampar and Teroi Rivers in Gunung Jerai Forest Reserve, Kedah. Source: Department of Irrigation and Drainage, Kedah.

This study was carried out in rivers of Gunung Jerai Forest Reserve (GJFR) in the state of Kedah, in the northern peninsular Malaysia (Fig. 1). At the height of 1217 m above sea level, Gunung Jerai (Kedah peak) (N5°47.44′E100°26.4′) has been deformed by granite plutonic intrusion and quartz porphyry (Chow 1980) bordering Kuala Muda and Yan districts. Eight rivers (streams) run down the peak of Gunung Jerai Forest Reserve within its catchment and three easily accessed rivers were selected for this study; Tupah River, Batu Hampar River and Teroi River. The physicochemical characteristics of the rivers: water depth, water temperature, velocity, pH and river width were measured using flow probe (Global Water, USA) for velocity and depths while pH and water temperature were measured using YSI Professional Plus Multi-Probe Meter Series 1550A (YSI Incorporated, USA).

### Tupah river

This second order river is located in Kuala Muda district. The 5.6 km long, 0.32±0.045 m of mean depth with mean width of 4.14±0.277 meter river entres Merbok River, which flows into the Straits of Malacca. The fast flowing Tupah River (0.56±0.157 m/s) has a yearly mean water temperature ranged from 22.8 to 25.7°C while the water pH ranging from 5.03 to 6.66. Tupah River composes of low land dipterocarp forest at 100–200 m above sea level. The substrates are predominantly cobble and gravel (55%), and the other 45% of river sediment is made up of boulder. This open canopy, clear water river is highly frequented by the locals during weekends and school holidays for various recreational activities. The sampling activities were done in this river at N5°45.008′ E100°26.526′.

### Batu Hampar river

The Batu Hampar River is located in Yan district. This 7.4 km long second order river flows through a populated village and fruit orchards in a low land dipterocarp forest at 300 m a.s.l. A moderately wide (4.73±0.375 m mean width) with 0.34±0.06 m mean depth river also entres the Straits of Malacca. The water flow is relatively fast (0.65±0.125 m/s) while pH of water ranges from 5.64 to 6.63. The annual mean water temperature ranges from 23.2 to 25.2°C. In the Batu Hampar River, the substrates such as cobbles and gravels were highly embedded (approximately 60%) into the bottom of the river. The locals visit this place during dry season, when the water flow is slower. The Batu Hampar River was developed by the Kedah state-government as a recreational park. Sampling activities for this study took place at N5°46.668′ E100°23.835′.

### Teroi river

This first order river is located high up on the Gunung Jerai at 1214 m in Gurun district. A shallow river (0.17±0.068 m mean depth) originated on Gunung Jerai peak has a mean width of 4.03±0.726 m. The water velocity of 1.22±0.123 m/s is the fastest among the three rivers because it flows over a steep slope. The water is acidic with pH values range from 4.06 to 6.21. The annual water temperature was between 19.1°C and 22.0°C. The Teroi River was partly shaded by tree canopies and flows through a hilly dipterocarp forest. Due to the resin of the *Agathis alba* trees that grow along the river bank, the colour of the water turns brownish. The sampling point was determined at N5°48.328′ E100°25.913′.

### Sampling Of EPT Immatures

Immatures of EPT were sampled from Tupah, Batu Hampar and Teroi Rivers, using a modified kick sampling technique of Merritt *et al.* (2008) beginning September 2007 until August 2008. A thorough descriptions of the sampling procedure can be accessed elsewhere (Suhaila & Che Salmah 2011). Twenty samples of aquatic insects was collected randomly at each river every consecutive months. The content of each sample or net was transferred into a transparent plastic bag, sealed, and sorted in the laboratory. Based on a preliminary sampling, 20 D-pond net samples collected more than 75% of the total insect taxa from each habitat. This was sufficient to represent EPT population (Radwell & Brown, 2007) that matched Elliot’s (1973) estimate of the population density for benthic study with a standard error of approximately 20%.

EPT nymphs and larvae were sorted visually and immersed in universal bottles containing 75% ethyl alcohol (ETOH). They were identified to respective genera under a dissecting microscope, Olympus CX41 (Olympus, Tokyo, Japan) using keys provided by Kenneth and Bill (1993), Morse *et al*. (1994), Wiggins (1996), Dudgeon (1999) and Yule and Yong (2004). The identifications of plecopterans were confirmed by Dr Ignec Sivec (Slovenian Museum of Natural History) and Professor Yeon Jae Bae from Korea University, Seoul, Korea verified the identifications of Ephemeroptera and Trichoptera.

### Water Samples Analysis

Five water samples (each) were collected from each river into a 500 mL polyethylene bottle monthly alongside the collections of the EPT. The bottles were kept in an ice chest and transported to the laboratory and preserved at 4°C until analysed. Ammonia-nitrogen (NH_3_-N) including other five water parameters; biochemical oxygen demand (BOD_3_), chemical oxygen demand (COD), total suspended solids (TSS), pH and dissolved oxygen (DO) were analysed. Dissolved oxygen (DO) content in the water and temperature were measured *in situ* using a YSI Model 550A (YSI Inc., Ohio, USA) oxygen meter while the water pH was measured with an electronic pH meter (HACH CO., Loveland, USA). The COD, TSS and NH_3_-N in the water were estimated in the laboratory using a standard kit of DR/890 HACH Calorimeter.

For measuring BOD_3_, water samples were confined at room temperature in the dark for 3 days. Before that, reading of DO was taken using the YSI Pro_BOD Probe (YSI Incorporated, USA) on the first day and another reading measured 3 days later after incubation. The difference between the first reading on the first day (DO_1_) and the second reading on the third day of incubation (DO_3_), was the amount of BOD_3_ in the water expressed in milligrams per liter.

### Water Quality Index Calculation

According to Malaysian Department of Environment (DOE 2002), to calculate the Water Quality Index (WQI) the means values of DO, BOD_3_, COD, pH, NH_3_-N and TSS were converted to sub-indices (SIs) (Appendix 1) using the best-fit equation and aggregated to compute the WQI according to the following equation:

WQI=0.22×SIDO+0.19×SIBOD+0.16×SICOD+0.15×SIAN+0.16×SISS+0.12×SIpH

where SI is the sub-index of each parameter.

### Statistical Analyses

Differences in mean monthly abundance of EPT in the three rivers were analysed using the Kruskal Wallis test at *p*=0.05 for non-normally distributed data (Kolgomorov-Smirnov test, *p*<0.05) using the SPSS software ver. 18. The total numbers of EPT taxa (genera) represented the EPT taxa Richness Index (Lenat 1993; Lenat & Penrose 1996) in each river. The relationship of the EPT taxa Richness Index with the WQI was assessed by Spearman’s Rho correlation and Regression analysis. The Canonical Correspondence Analysis (CCA) of CANOCO program version 4.0, investigated the influence of water quality parameters on the distribution and abundance of EPT taxa in the rivers. The Monte-Carlo test was applied to test the significance of the produced canonical axes with 499 permutations at *p*<0.05. The biplot ordination diagram was produced using the CanoDraw for Windows 4.1.

## RESULTS

The result showed that Tupah River recorded the highest EPT taxa Richness index with 28 taxa, followed by Batu Hampar River (25) and Teroi River with 22 taxa (Table 1). The 28 genera found in all rivers were represented by varied composition patterns. Among the 12 ephemeropterans genera encountered, *Baetis* spp. was the most common genus with its greatest abundance occurred in Teroi River and the least in Batu Hampar River. It was followed by *Platybaetis* spp., which was more abundant in Teroi River but only few of them were found in Batu Hampar River. *Thalerospyrus* spp. was the third most common genus of Ephemeroptera. This genus had low mean abundances in both Tupah River and Batu Hampar River, much lesser than the number recorded in Teroi River.

Two Ephemeroptera genera; *Caenis* spp. and *Habrophlebiodes* spp. collected from Tupah and Batu Hampar Rivers were absent from Teroi River. *Isonychia* spp. was only found in Batu Hampar River. Plecopterans *Etrocorema* spp. and *Cryptoperla* spp. were not encountered in Teroi River. Two Trichoptera genera (*Ganonema* spp. and *Lepidostoma* spp.) were not collected from Teroi River and two others (*Rhyacophila* spp. and *Marilia* spp.) were not found in both Tupah and Batu Hampar River.

Six genera of Plecoptera were recorded and *Neoperla* spp. was the most abundant genus in the three rivers. The highest mean abundance of *Neoperla* spp. was found in Batu Hampar River and the least in Teroi River. It was followed by *Phanoperla* spp., which was also more abundant in Batu Hampar River and the fewest in Teroi River. *Kamimuria* spp. had higher mean abundances in both Tupah River and Batu Hampar River but was lower than that obtained from Teroi River. Among the trichopterans, *Cheumatopsyche* spp. was the most common genus. Its mean abundance was the highest in Tupah River and the least in Teroi River.

From the biological perspective (Table 2), the EPT taxa Richness index indicated that the habitats (as well as water quality) in all selected rivers were classified into a non-impacted category as all rivers collected more than 10 taxa of EPT (USEPA 1990). However, the EPT collected from the three rivers were highly variable in compositions and total number of individuals collected. Lenat’s (1993) classification of water quality classified Tupah and Batu Hampar rivers into good quality while Teroi River fell into good-fair category. Many EPT taxa collected from Tupah River inhabited Batu Hampar River but fewer common taxa were found in Teroi River.

Composition of EPT communities were much higher in Batu Hampar River (H’=2.29) followed by Tupah River (H’=2.15) then Teroi River (H’=0.77) (Table 3). A similar pattern was shown by scores of Simpson Diversity Index (1-D). Batu Hampar River (1-D=0.85) and the Tupah River (1-D=0.81) scored high but low in Teroi River (1-D=0.32). Species richness for the Menhinick Index (R) was low at all rivers, ranging from 0.43 to 0.19. Based on these values, Tupah River provided the most suitable habitats for the EPT community compared to the other two rivers.

The EPT distribution based on Pielou Evenness Index was more uniform in Batu Hampar (E = 0.71) and Tupah rivers (E = 0.65), least evenly distributed in Teroi River (E = 0.26). The Kruskal-Wallis test revealed there was a significant difference between indices with the rivers (Shannon-Wiener index, χ^2^ = 48.765, P = 0.00; Simpson’s index, χ^2^ = 31.919, P = 0.00; Pielou index, χ^2^ = 72.504, P = 0.00; Menhinick index, χ^2^ = 11.075, P = 0.00).

**Figure 2:**
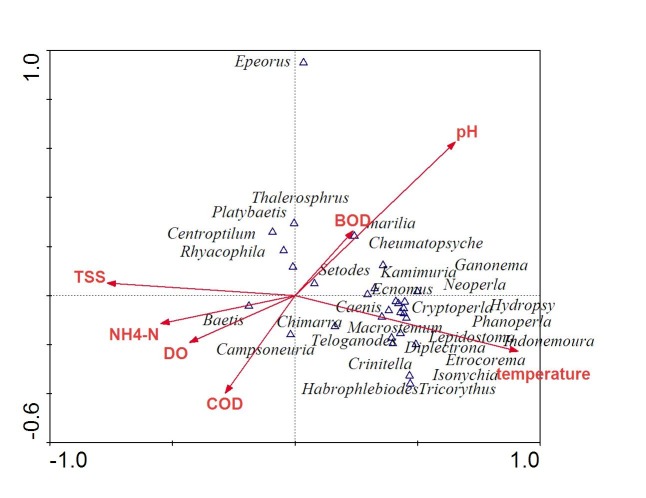
First two axes from canonical correspondence analysis (CCA) of Ephemeroptera, Plecoptera and Trichoptera genera and environmental parameters in the Tupah, Batu Hampar and Teroi Rivers, Kedah.

All rivers were categorised into five classes of water quality categories by the Water Quality Index (WQI) of the Malaysian Department of Environment (DOE 2002) which focuses on water uses (Table 4). The WQI scores ranged from 80.41 to 93.14 with the highest value recorded in Teroi River. The score in Teroi River represents a Class I river which indicates very clean water, readily consumable by human. Tupah River (84.28) and Batu Hampar River (80.41) were categorised into Class II implying good water quality, suitable for human consumption but requires minimal treatment. There was no correlation between the EPT taxa Richness Index and WQI (*r* = 0.021, *p* = 0.452) and low dependence of the scores of both indices was exhibited in Figure 2 based on Canonical analysis.

According to CCA analysis, certain parameters have influenced the distribution and abundance of the EPT taxa (Table 5). The total extent of variation or total inertia (TI=1.344) encountered for 45.8% of the variance contributed by the constrained eigenvalues (TVE = Total Variance Explained) of seven measured variables. Seventy point one percent (70.1%) of the variance in species–environmental relationships was contributed by its variables in the first axis and 12.6% of the variance was accounted for the second axis. The Monte Carlo test was significant for all axes at *P* < 0.05. The t-value biplot distribution of *Etrocorema* spp., *Lepidostoma* spp.*, Hydropsyche* spp.*, Diplectrona* spp. and *Chimarra* spp. are characterized by higher water temperature. High pH value was likely to influenced *Cheumatopsyche* spp. while *Marilia* spp. and *Thalerosphyrus* spp. were affected by high BOD_3_ content. Low COD content characterised the distribution of *Centroptilum* spp.*, Rhyacophylia* spp.and *Platybaetis* spp.

**Figure 3:**
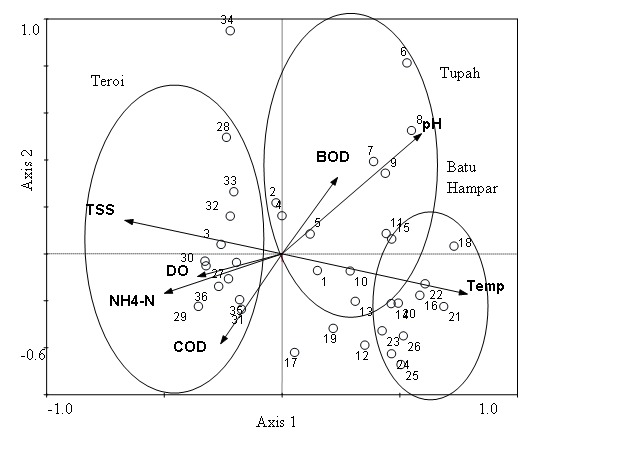
The first two axes of principal correspondence analysis (PCA) of the rivers; Tupah River (1–13), Batu Hampar River (14–26), Teroi River (27–39) in the Gunung Jerai Forest Reserve, Kedah.

The CCA diagram showed obvious variation in spatial pattern of the EPT assemblages when the samples collected from all rivers were plotted (Fig. 3). Tupah River (points 1–12) was distinguished with high diversity of Trichoptera and characterised by high contents of biochemical oxygen demand (BOD_3_) compared to other rivers. The Tupah and Batu Hampar rivers (points; 1–12, 13–24, respectively) showed slight overlapping as they shared some similar conditions such as the BOD_3_ and pH contents in the water. The samples from Teroi River clearly separated (points 25–36) from other rivers which characterised by having higher DO, COD and NH_3_-N and low pH contents in the water.

## DISCUSSION

Although all selected rivers supported almost equally rich fauna, the diversity and abundance of the EPT were significantly different among rivers. The assemblages and occurrence of EPT in each river depends on river physical and its microhabitats. However, the scores of EPT taxa Richness fell far above the limit for non-impacted water quality (EPT taxa Richness Index = 10) (USEPA 1990); 28 taxa in Tupah River, 25 taxa in Batu Hampar River and 22 taxa in Teroi River suggesting all rivers were minimally or undisturbed and remained in good condition. Based on Lenat’s (1993) classification using the EPT taxa Richness Index in hill country streams, the water quality in Tupah and Batu Hampar rivers were in good condition (EPT = 24–31) while in Teroi River the water was in Good-Fair category (EPT = 16–23). Classification of water quality using the EPT taxa Richness Index in this study is considered satisfactory because according to Lenat (1993), the EPT reach their maximum development in the headwaters and consists of families that are restricted or belong to clean running water. Moreover the values of this index are sensitive and changes following the change in river water quality (Dudgeon 1996). EPT is important to be use in upstream rivers as their abundance and diversity was useful as reference for interpreting biological index. Furthermore, EPT species have limited levels of tolerance to any changes in the river water and this action can be used in detecting environmental perturbations. In contrary, the scores of measured chemical water parameters (WQI of the Malaysian Department of Environment 2002) showed that Teroi River had excellent water quality of Class I (93.14) and Tupah as well as Batu Hampar rivers were categorised into Class II (84.28 and 80.41, respectively) of good water quality. The WQI classified Tupah and Batu Hampar rivers into similar category but Teroi River was two classes above the classification of the EPT taxa Richness Index assuming the river categories represented identical quality of water.

Having shown that the WQI classified Teroi River as having excellent water quality (Class I). The composite value of six water quality parameters used in calculation of the WQI indicated the least disturbed environment in Teroi River although the water was acidic. Typically, the benthic communities in Class I (pristine) river respond only to inert pollutants (Ogbeibu & Victor 1989). Presently, river degradation causes marked reduction in faunal composition. Atrophic influence is one of the factors affecting composition of aquatic insects (Rosenberg & Resh, 1993). In disturbed rivers, a decrease of sensitive taxa and an increase of tolerant taxa are expected. The biological integrity of Teroi River was relatively unimpacted and the EPT populations were generally in its best condition as represented by its relatively high EPT taxa Richness Index. However, the EPT taxa Richness Index score in Teroi River was lower than in Tupah and Batu Hampar rivers possibly related to low pH of the water in Teroi River which was less suitable to some of the EPT genera. Nevertheless, categorisation of river class (water quality) based on single parameter by the National Interim Water Quality Standards (DOE 2002) assigns the river with such water pH (4.06 to 6.21) into Class III, similar to the EPT taxa Richness Index categorisation of the river (Good-Fair). In this case the EPT taxa Richness Index is very sensitive in detecting the quality of the water compared to the WQI although only one of WQI’s parameter falls in the range of poorer quality.

For biological organisms, the increasing acidity of water obviously affected in decreasing of organic matter decomposition (Burton *et al.* 1985) and a decrease in macroinvertebrate’s diversity (Okland & Okland 1986, Winterbourn & Collier 1987, Suhaila & Che Salmah 2014). Changes in macroinvertebrate assemblages that changed the predator-prey relationships are coincided with the acidification of the water (Bendell & Macnicol, 1987; Eriksson *et al*. 1980) especially the Ephemeroptera that survive well in an acidic environment (Dangles & Guerold 2000). Similarly, ephemeropterans Baetidae and Heptageniidae proliferated in Teroi River but the diversity of EPT in this river was lower compared to two other rivers.

In this study, *Baetis* spp. and *Platybaetis* spp. (Ephemeroptera) showed high tolerance to wide pH range, from acidic to neutral (4.90 to 7.0). Based on the CCA biplot, the most abundant taxa in Teroi River were *Baetis* spp. and *Platybaetis* spp. and indicated preference to low pH water. Gerhadt *et al*. (2005) stated that mayflies are able to survive in acidic water with pH as low as 4.5. According to Rosemond *et al.* (1992), in Great Smoky Mountains National Park, Tennesse, USA, the ephemeropteran *Ameletus lineatus* Traver (family Siphonoluridae) was found more abundant in a more acidic water (pH 4.5 to 6.4) compared to other species. Nevertheless, *Baetis* spp. is present in high abundances in many rivers due to its active oviposition, which in turn depends largely on the availability of egg deposition sites (Elliott 1972; Bengtsson 1988). Peckarsky *et al.* (2000) noticed an increase in egg deposition by *B.bicaudatus w*hile working in a high-altitude river and advocated that this is the result of increased number of rock substrates protruding from the water surface. Although bedrock formed the substrate of Teroi River, high abundance of *Baetis* spp. implied that this river had rock surfaces suitability as oviposition sites for this species.

Increasing pH values (neutral) which were recorded from Tupah and Batu Hampar rivers negatively correlated with plecopterans such as *Neoperla* spp. and *Cryptoperla* spp. and *Cheumatopsyche* spp. (Trichoptera). Thus, the absence of caenids, leptophlebiids and oligoneurids in Teroi River implicated that these genera were not able to withstand acidic water. Other ephemeropterans in Teroi River such as *Thalerosphyrus* spp., *Campsoneuria* spp., *Epeorus* spp., *Baetis* spp., *Platybaetis* spp*.*, *Centroptilum* spp*.*, *Tricorythus* spp., *Teloganodes* spp. and *Crinitella* spp. which were also found in other rivers, tolerated wide range of water pH.

The contents of COD and NH_3_-N were relatively low in all rivers. Although slightly higher values of these parameters were recorded in Teroi River, this level did not exceed the Class II limit of the Malaysian WQI thus their influence on EPT diversity seemed negligible. Among the three rivers, Teroi River had a higher NH_3_-N content in the water which probably caused by animals’ excreta especially from the large population of monkeys (long-tailed and pig-tailed macaques) found in the river’ surroundings. Only trichopterans *Hydropsyche* spp. and *Macrostemum* spp. negatively correlated with NH_3_-N content and they were well represented in Batu Hampar River. Tupah and Batu Hampar Rivers had low NH_3_-N contents showing that both rivers are having good water quality.

*Kamimuria* spp. and *Neoperla* spp. from family Perlidae breath through external gills on their thoraxes and abdomens. The gills are located along the body, effectuating this family dependence on high dissolved oxygen in the water to respire thus influences distribution of *Kamimuria* spp. in rivers as observed in this study. On the other hand, the amount of BOD in the water charcterized the distribution of *Phanoperla* spp. (Plecoptera) and *Cheumatopsyche* spp. (Trichoptera). The water body with high levels of organic matters relatively has higher content of BOD (Thani & Phalaraksh 2008). The highest BOD_3_ content (1.93 mg/L) was recorded in Tupah River because besides forest debris, other organic matters could be introduced by high number of visitors in the area. However, the values recorded in Tupah River did not exceed the upper limit of Class II of WQI of Malaysia (DOE 2002).

Furthermore, Tupah and Batu Hampar rivers had lower TSS content compared to Teroi River which implicates the water column in these rivers had less suspended particles. Clean clear water may have positive effects on food availability as sunlight penetration can increase photosynthesis process in algae and macrophytes. This appropriate condition favours the EPT community resulting more diverse taxa.

The status of water quality in all rivers from the WQI and EPT taxa Richness Index classifications were further supported by the results of the canonical corresponding analysis (CCA) which compared the physico-chemical data with the diversity of EPT. The three rivers were classified into two groups. The first group which includes Tupah and Batu Hampar Rivers, has a good quality of water. Teroi River made the second group with excellent water quality (WQI) but it has lesser EPT taxa. The separation of rivers following the distribution of EPT taxa in this study correlated strongly with the quality of water of the WQI. They both separated the rivers based on shared water quality. This is obvious when the WQI classified the water quality in Teroi River into Class I while the EPT taxa Richness Index assigned it into good-fair category which is equivalent or more or less similar to Class III of the WQI. It is best to note that the WQI classification focuses on quality of water using values of physico-chemical water parameters strictly for human uses and completely ignores the tolerances of aquatic organisms towards its selected parameters.

## CONCLUSION

The EPT taxa Richness Index is sensitive to environmental impairment as EPT taxa are potentially sensitive to changes (especially increasing disturbances) in various headwater water parameters. Therefore using the EPT taxa Richness Index is a relatively accurate biological parameter to detect aquatic disturbances although more investigation is required to further improve the application of the index. The survival of EPTs depends strongly on the good ranges of the river physico-chemical environment that indirectly indicates healthy river status, hence increases reliability of water quality assessment especially in pristine environments. This study indicated that the EPT taxa Richness Index is more effective than the WQI in classifying the river water quality in GJFR.

## Figures and Tables

**Table 1:** Mean abundance (ind/m^2^ ± standard error) of Ephemeroptera, Plecoptera and Trichoptera in rivers of Gunung Jerai Forest Reserve, Kedah.

Order	Family	Genus	Tupah River	Batu Hampar River	Teroi River
Ephemeroptera	Caenidae	*Caenis*	6.08 ± 0.45	4.17 ± 0.7	0
	Leptophlebiidae	*Habrophlebiodes*	4.33 ± 2.13	5.75 ± 2.53	0
	Baetidae	*Baetis*	12.8 ± 4.5	57.7 ± 3.8	610.1 ± 9.9
		*Platybaetis*	19.2 ± 5.2	5.7 ± 1.4	49.6 ± 24.4
		*Centroptilum*	1.83 ± 0.34	0.25 ± 0.13	3.42 ± 0.73
	Teloganodidae	*Teloganodes*	0.25 ± 0.13	0	0.92 ± 0.07
	Tricorythidae	*Tricorythus*	4.08 ± 2.13	5.25 ± 2.65	0
	Heptageniidae	*Thalerosphyrus*	14.9 ± 7.2	13.3 ± 2.1	51.1 ± 14.8
		*Campsoneuria*	1.25 ± 0.39	1.92 ± 0.9	8.33 ± 0.41
		*Epeorus*	2.75 ± 0.77	1.0 ± 0.3	2.42 ± 0.84
	Oligoneuridae	*Isonychia*	0	0.33 ± 0.86	0
	Ephemerellidae	*Crinitella*	1.25 ± 0.6	0	0.17 ± 0.01
Plecoptera	Perlidae	*Phanoperla*	2.75 ± 0.57	4.58 ± 0.84	0.67 ± 0.02
		*Neoperla*	43.8 ± 5.1	67.9 ± 6.1	2.8 ±0.8
		*Kamimuria*	3.42 ± 0.51	1.25 ± 0.3	0.92 ± 0.31
		*Etrocorema*	0.92 ± 0.22	0.17 ± 0.01	0
	Peltoperlidae	*Cryptoperla*	1.75 ± 0.43	1.17 ± 0.37	0
	Nemouridae	*Indonemoura*	0.83 ± 0.3	0.92 ± 0.31	0.25 ± 0.13
Trichoptera	Hydropsychidae	*Hydropsyche*	15.1 ± 6.9	30.1 ± 1.0	1.4 ± 0.4
		*Macrostemum*	9.5 ± 3.46	3.17 ± 0.73	1 ± 0.35
		*Cheumatopsyche*	68.4 ± 2.9	32.2 ± 1.2	2 ± 0.6
		*Diplectrona*	4.67 ± 0.92	5.58 ± 1.63	1.08 ± 0.43
	Ecnomidae	*Ecnomus*	0.5 ± 0.26	3.08 ± 0.63	0.92 ± 0.36
	Calamoceratidae	*Ganonema*	0.33 ± 0.14	0.8 ± 0.08	0
	Rhyacophilidae	*Rhyacophila*	0.83 ± 0.27	0	1 ± 0.3
	Lepidostomatidae	*Lepidostoma*	0.67 ± 0.31	2.08 ± 0.57	0
	Philopotamidae	*Chimarra*	12.9 ± 1.2	19.2 ± 5.2	3.5 ± 0.6
	Leptoceridae	*Setodes*	0.17 ± 0.11	0.5 ± 0.19	0.5 ± 0.1
	Odontoceridae	*Marilia*	0.33 ± 0.19	0	0.17 ± 0.11

**Table 2:** Biological indices based on composition and abundance of EPT immature and water quality evaluation in selected rivers of Gunung Jerai Forest Reserve, Kedah.

River	EPT taxa richness index	Water quality assessment
Tupah	28	Non-impacted
Batu Hampar	25	Non-impacted
Teroi	22	Non-impacted

**Table 3:** Evaluation of EPT abundance and diversity using Shannon-Wiener Index (H’), Simpson’s Index (1-D) and Menhinick Index (R), Pielou Evenness Index (E) for Tupah, Batu Hampar and Teroi rivers, Kedah.

River	Shannon-Wiener Index (H’)	Simpson’s Index (1-D)	Menhinick Index (R)	Pielou Index (E)
Tupah	2.153	0.811	0.427	0.646
Batu Hampar	2.294	0.853	0.432	0.713
Teroi	0.765	0.323	0.193	0.260

**Table 4:** Mean values of water parameters and classification of sampling sites in rivers of Gunung Jerai Forest Reserve based on the WQI (DOE 2002).

Parameter	Tupah	Batu Hampar	Teroi
DO (mg/L)	7.53 ± 0.22	7.14 ± 0.37	7.67 ± 0.33
SIDO	97.07	89.71	94.29
BOD (mg/L)	1.93 ± 0.07	0.95 ± 0.18	0.84 ± 0.18
SIBOD	92.24	96.38	96.85
COD (mg/L)	10.25 ± 0.23	10.32 ± 1.91	19.15 ± 3.15
SICOD	85.47	85.37	73.63
NH_3_-N (mg/L)	0.02 ± 0.01	0.03 ± 0.01	0.04 ± 0.04
SIAN	98.4	97.35	95.3
TSS (mg/L)	2.85 ± 0.23	1.46 ± 0.31	5.23 ± 0.21
SISS	37.63	19.31	69.23
pH	6.02 ± 0.12	6.06 ± 0.11	4.97 ± 0.21
SIPH	91.2	91.78	56.72
WQI	84.28	80.41	93.14
Class	II	II	I

**Table 5:** Correlations, eigenvalues and variance explained for the first two axes of canonical correspondence analysis (CCA) for Ephemeroptera, Plecoptera and Trichoptera larvae abundance (organisms/ samples) and environmental parameters for all dates sampled for Tupah, Batu Hampar and Teroi Rivers from Gunung Jerai Forest Reserve, Kedah.

Variable	Axis 1	Axis 2	Total inertia
pH	0.5755	0.4664	
Temp (°C)	0.7514	−0.1802	
DO (mg/L)	−0.3573	−0.0621	
BOD_3_ (mg/L)	0.2163	0.2731	
COD (mg/L)	−0.2491	−0.3605	
TSS (mg/L)	−0.6440	0.0960	
NH_3_-N (mg/L)	−0.4680	−0.1195	
Eigenvalues:	0.431	0.078	1.344
Species-environment correlations:	0.843	0.813	
Cumulative percentage variance of:			
species data	32.1	37.9	
species-environment relation	70.1	12.6	
Sum of all eigenvalues			1.344
Sum of all canonical eigenvalues			0.616
Total variance explained (TVE):			45.8%

Summary of Monte Carlo test

Test of significance of first canonical axis: eigenvalue=0.431, F-ratio=14.659, P-value=0.002

Test of significance of all canonical axes: Trace=0.616, F-ratio=3.746, P-value=0.002

## APPENDIX

### Appendix 1 Best Fit for the estimation of the various sub-index values (Department of Environment, 2002)

**Table:**

Subindex for DO (in % saturation)	
SIDO = 0	for x ≤ 8
= 100	or x ≥ 92
SIDO = −0.395 + 0.030x^2^ − 0.00020x^3^	for 8 < x < 92
Subindex for BOD	
SIBOD = 100.4 – 4.23x	for x ≤ 5
SIBOD = 108* exp (−0.0157x − 0.04x)	for x > 5
Subindex for COD	
SICOD = −1.33x + 99.1	for x ≤ 20
SICOD = 103*exp (−0.0157x −0.04x)	for x ≥ 20
Subindex for AN	
SIAN = 100.5 – 105x	for x ≤ 0.3
SIAN = 94* exp (−0.573x – 5* |x-2|)	for 0.3 <x<4
SIAN = 0	for x ≥ 4
Subindex for SS	
SISS = 97.5* exp (−0.0067x + 0.05x)	for x ≤ 100
SISS = 71* exp (−0.001x – 0.015x)	for 100 < x < 1000
SISS = 0	for x ≥ 1000
Subindex for pH	
SIPH = 17.2 – 17.2x + 5.02x^2^	for x < 5.5
SIPH = −242 + 95.5x −6.67x^2^	for 5.5 ≤ x < 7
SIPH = −181 + 82.4x – 6.05x^2^	for 7 ≤ x < 8.75
SIPH = 536 – 77.0x + 2.76x^2^	for x ≥ 8.75

(x = concentration in mg/L for all parameters except pH and DO)

Aggregate the calculation according to the following equation:

WQI=0.22×SIDO+0.19×SIBOD+0.16×SICOD+0.15×SIAN+0.16×SISS+0.12×SIPH

Where SI is the subindex of each parameter. Based on the WQI values, the water quality is categorized.
